# Predicting explainable dementia types with LLM-aided feature engineering

**DOI:** 10.1093/bioinformatics/btaf156

**Published:** 2025-04-08

**Authors:** Aditya M Kashyap, Delip Rao, Mary Regina Boland, Li Shen, Chris Callison-Burch

**Affiliations:** Department of Computer and Information Science, University of Pennsylvania, Philadelphia, PA 19104, United States; Department of Computer and Information Science, University of Pennsylvania, Philadelphia, PA 19104, United States; Department of Mathematics and Data Science, Saint Vincent College, Latrobe, PA 15650, United States; Department of Biostatistics, Epidemiology and Informatics, University of Pennsylvania, Philadelphia, PA 19104, United States; Department of Computer and Information Science, University of Pennsylvania, Philadelphia, PA 19104, United States

## Abstract

**Motivation:**

The integration of Machine Learning and Artificial Intelligence (AI) into healthcare has immense potential due to the rapidly growing volume of clinical data. However, existing AI models, particularly Large Language Models (LLMs) like GPT-4, face significant challenges in terms of explainability and reliability, particularly in high-stakes domains like healthcare.

**Results:**

This paper proposes a novel LLM-aided feature engineering approach that enhances interpretability by extracting clinically relevant features from the Oxford Textbook of Medicine. By converting clinical notes into concept vector representations and employing a linear classifier, our method achieved an accuracy of 0.72, outperforming a traditional n-gram Logistic Regression baseline (0.64) and the GPT-4 baseline (0.48), while focusing on high-level clinical features. We also explore using Text Embeddings to reduce the overall time and cost of our approach by 97%.

**Availability and implementation:**

All code relevant to this paper is available at: https://github.com/AdityaKashyap423/Dementia_LLM_Feature_Engineering/tree/main.

## 1 Introduction

A tremendous need exists for Machine Learning (ML) and Artificial Intelligence (AI) algorithms in the healthcare domain. Healthcare is one of the fastest-growing fields in terms of producing data, outgrowing even the financial services industry (Hong *et al.* 2018). With all of this data generated daily by clinicians, nurses, radiologists, and other healthcare providers, there is a need for more robust algorithms that can learn novel patterns from these diverse, multi-modal data. However, using out-of-the-box AI models is not desirable due to their traditional lack of explainability and transparency in terms of statistical decision-making (Schwartz *et al.* 2024). Without transparency, clinicians and other healthcare practitioners are unsure as to whether they can trust, and ultimately implement such algorithms.

### 1.1 Importance of model explainability and limitations of existing large language models

Ensuring model explainability and correct chain of reasoning is an integral part of the clinical development pipeline that can help promote trust among the end users, namely the physicians and the patients. Explainable models allow physicians to understand why a prediction was made in a clinically relevant fashion. Neural Networks, which are often used for clinical outcome modeling, have been shown to have high accuracies but their predictions are often opaque, depend on shallow heuristics (McCoy *et al.* 2019), and are treated as black-boxes (Schwartz *et al.* 2024). In this article, we argue that it is preferable to have a model that is both explainable and accurate.

Numerous factors constrain the application of Neural Network approaches. For instance, McCoy *et al.* (2019) showed that although Statistical Neural Network (SNN) models like BERT (Devlin *et al.* 2018) achieved high scores on different tasks, they did so by learning superficial rules that work for most training instances, rather than learning the fundamental principles of the task. For example, in a natural language inference task which involves determining whether one sentence entails another, BERT failed to correctly interpret differences between subject-object relationships for sentence pairs such as “The doctor saw the lawyer.” and “The lawyer saw the doctor.”, highlighting their tendency to rely on shallow heuristics rather than a comprehensive understanding of language. Given BERT’s limitations in handling such tasks, it can be inferred that its variants such as BioBERT (Lee *et al.* 2020) and ClinicalBERT (Alsentzer *et al.* 2019) would similarly struggle to effectively capture the complex inter-dependencies between patient risk factors required in clinical diagnosis.

Some of the more recent SNN models like ChatGPT, GPT-4, and other Large Language Models (LLMs) have been additionally shown to hallucinate when insufficient information is provided (Chen *et al.* 2023, Liu *et al.* 2023, Shen *et al.* 2023). They also tend to reproduce biases and factual inconsistencies in their training data (Liu *et al.* 2023, Shen *et al.* 2023). These issues are especially pronounced for the health care field where accuracy of information is crucial. Sandmann *et al.* (2024) evaluated the clinical accuracy of GPT-3.5 and GPT-4 on 110 medical cases across diverse clinical disciplines for suggesting initial diagnosis, examination steps, and treatment. They suggest not using these models without medical experts as they lack consistent high accuracies across different tasks and perform poorly on rare diseases. Another study (Cascella *et al.* 2023) found that ChatGPT was unable to address causal relationships between acute respiratory distress syndrome and septic shock. They argue that since ChatGPT was not designed to answer medical questions, it lacks the medical expertise and context needed to fully understand the complex relationships between different conditions and treatments.

### 1.2 Techniques and challenges in interpretable AI for healthcare

Despite the promising performance of Deep Learning networks in various domains, their adoption in healthcare has been slow. This is due to the fact that more insight into the inner workings of AI tools is required for it to become clinically successful (Salahuddin *et al.* 2022). Unlike consumer applications like ChatGPT, medical applications have a higher bar for approval and are governed by strong regulatory compliance laws (Temme 2017). While LLMs have shown impressive performance, some of the most popular models like ChatGPT and GPT-4 are not open source, limiting transparency and the ability of researchers to mitigate biases and hallucinations (Wang *et al.* 2023).

Interpretability and explainability have often been used synonymously in the field of explainable AI (Chakraborty *et al.* 2017, Adadi and Berrada 2018) and have four primary dimensions along which they are defined (Luo *et al.* 2024): (i) Faithfulness: Measures how well an interpretation method describes the decision-making process used by the underlying model. (ii) Stability: An interpretation method that provides similar explanations for similar inputs is considered stable. (iii) Comprehensability: Measures how understandable an interpretation to an end-user is. (iv) Trustworthiness: Interpretations can be called trustworthy only when the experiment design is carefully considered, in that their results can only be relied upon if carefully controlled.

Researchers have identified important input features that significantly impact a model’s prediction using different techniques. Rationale Extraction involves extracting phrases from the original textual inputs that represent critical features that influence the output (Li *et al.* 2018, Bastings *et al.* 2019, Zellers *et al.* 2021). Input perturbations modify or remove up to a few words in the original input to measure a performance change (Basaj *et al.* 2018, Rudin 2018, Ribeiro *et al.* 2020, Yordanov *et al.* 2021). Alternatively, attention weights which are weighted sum scores of the input representation in the intermediate layers of neural networks are measured (Yu 2013, Bahdanau *et al.* 2014, Luong *et al.* 2015, Vedantam *et al.* 2015). Finally, attribution methods examine the gradients of a model to identify the most important features (Hendricks *et al.* 2018, Tutek and Šnajder 2018). Other approaches generate Natural Language Explanations in addition to the outcome of interest (Camburu *et al.* 2018, Rajani *et al.* 2019, Kumar and Talukdar 2020). Camburu *et al.* (2019) showed that some of the explanations with these approaches were inconsistent with the predicted outcomes, hinting at either an incorrect explanation or a flawed model decision-making process.

In this article, we draw inspiration from Concept Bottleneck Models (CBMs) (Koh *et al.* 2020) to develop an LLM-aided feature engineering pipeline for patient dementia-type prediction using their clinical notes. Rather than have a Neural Network perform end-to-end classification as done traditionally, CBMs rely on the idea of generating high-level task-related concepts during training time, and then using these concepts to predict a label. For example, a bird identification task might involve high-level concepts such as *“beak length”* or *“wing color*,*”* while for a knee X-ray grading task, concepts could include *“bone spurs”* or *“narrow joint space*.*”* Finally, a simple linear model is trained on these intermediate concepts, and the most important features are obtained by examining the model weights. Mo *et al.* (2024) used LLMs to generate interpretable linguistic markers for Alzheimer’s disease detection, where the extracted features improved model interpretability even if accuracy was not significantly enhanced. McInerney *et al.* (2023) introduced Crafting High-Level Latents (CHiLL), where LLMs are prompted with carefully designed queries created by experts to extract meaningful features from clinical notes. Their results showed that linear models using features from CHiLL performed comparably to those using traditional reference features such as ICD codes while also providing better interpretability than those relying on “Bag-of-Words” features, which are less informative. Additionally, their study found that weights assigned to the features learned by the linear model aligned well with clinical expectations, indicating that their method not only extracts useful features but also maintains relevance to clinical practice.

### 1.3 Urgent need for an interpretable prediction model for Alzheimer’s disease and related dementias

Alzheimer’s Disease and Related Dementias (ADRD) is a group of related conditions that consist of progressive neurodegeneration that typically presents initially with forgetfulness and confusion (Atri 2019, Tahami Monfared *et al.* 2022). ADRD currently afflicts 6 million patients in the USA, with projections estimating 13 million by 2050 (https://www.alz.org/alzheimers-dementia/facts-figures). Dementia collectively kills more patients per year than breast and prostate cancers combined. However, despite its frequency of incidence, little is known about patients in community-based settings as derived from Electronic Health Records (EHR) cohorts. This is because the majority of ADRD research focuses on postmortem (after the patient has died) samples or expensive clinical trials (that often lack racial and ethnic diversity). There remains a paucity of ADRD research among diverse populations, including investigating sex disparities (Tang *et al.* 2022), and racial disparities (Babulal *et al.* 2019) in outcomes. Additionally, many state-of-the-art studies on ADRD have limited generalizability due to a lack of racial, ethnic, and socioeconomic inclusiveness (Chin *et al.* 2011).

Development of an AI model that is able to predict ADRD development among patients being seen in a hospital, including the subtype of dementia (e.g. Alzheimer’s Disease, vascular dementia, or other dementias) among patient cohorts derived from real-world clinical EHR cohorts would fill a tremendous and urgent need. However, many of the current models, like the LLMs previously discussed, are not interpretable and lack model explainability that would enable them to be translated from the AI bench to the clinical bedside and thereby enable utility in real-world settings. Development of explainable AI models remains a critical unmet need in biomedical research (Payrovnaziri *et al.* 2020, Xu *et al.* 2023b).

### 1.4 Our proposed model to predict ADRD type and overcome LLM limitations

We keep in mind the limitations discussed in Section 1.2 and formulate a task for predicting the type of dementia that a patient has using clinical notes from their EHRs. We created an LLM-aided feature engineering pipeline that ensures the model looks at clinical features most relevant to the outcome of interest, thereby minimizing the likelihood of spurious correlations and hallucinations in the prediction process.

We provide a novel method for extracting clinically relevant features using the Oxford Textbook of Medicine (Warrell 2003). This allows us to extract patient features related to dementia from their clinical notes. An example of an extracted feature using our approach is *“The patient exhibits poor memory.”* which is a human-readable sentence making our approach interpretable. In order to predict the dementia type of patients, we first convert their clinical notes into a concept vector representation. Next, we train a linear classifier on this representation to predict patient dementia type. Finally, we analyze the weights of the linear model to provide insights into the models decisions as well as the most important clinical features for dementia type prediction.

The contributions of this article are as follows:

1. We develop a novel method of extracting patient clinical features related to dementia from the Oxford Textbook of Medicine (Warrell 2003).
2. We develop an LLM-aided feature engineering pipeline that converts patient notes into concept vector representations, which we then use to train a linear interpretable model on top to analyze important features. Our approach obtains an accuracy of 0.72 on a held-out test-set which beats an ngram Logistic Regression baseline (0.64) and a GPT-4 baseline (0.48) while being more interpretable.

## 2 System design

**Figure 1.**
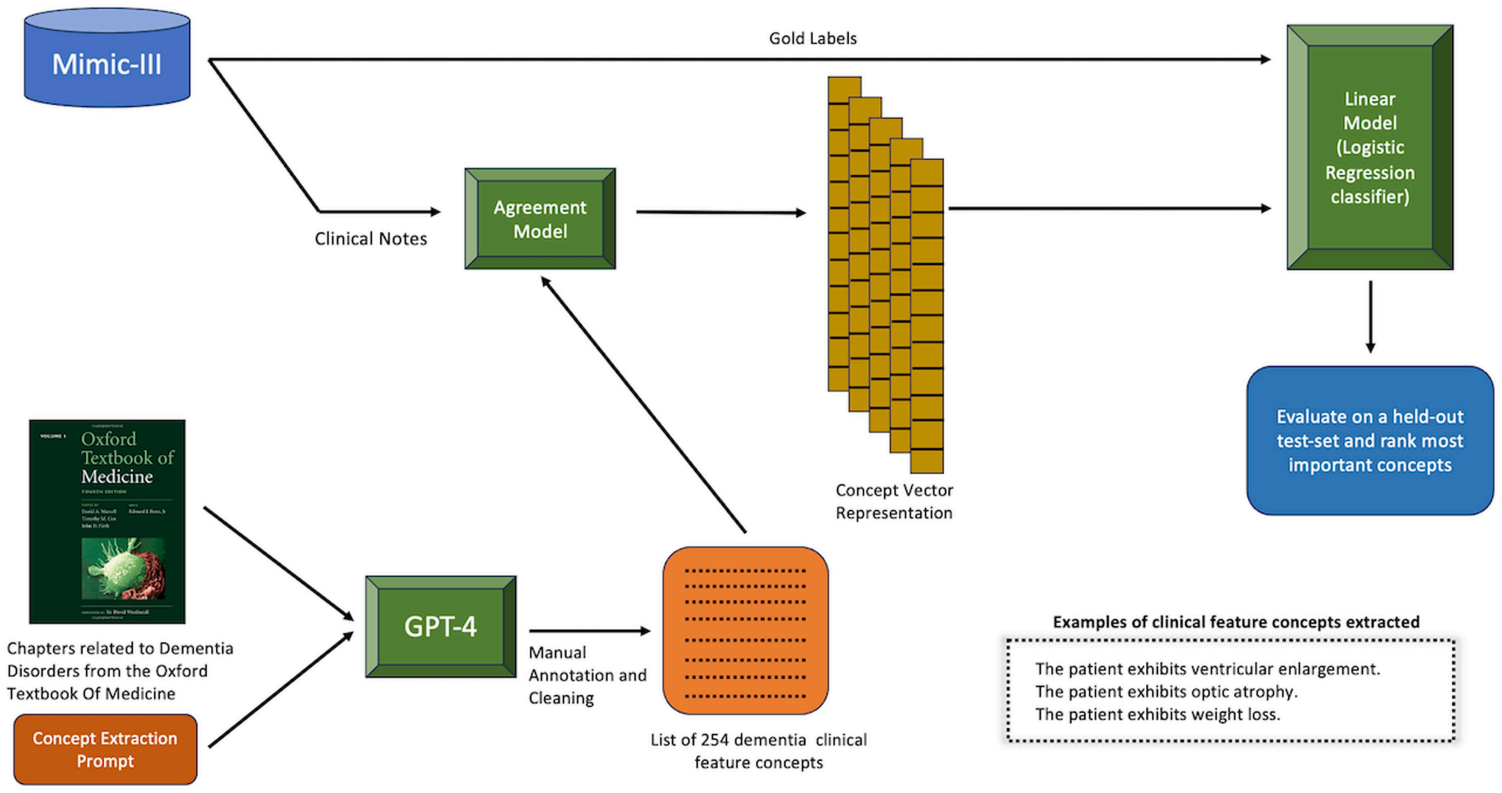
An overview of dementia-type classification with patient clinical notes using our LLM-aided feature engineering approach.

Our goal is to design an explainable clinical decision pipeline that predicts patient dementia type using clinical notes from their EHRs. An illustration of the overall pipeline is shown in Fig. 1. At a high level, we first use GPT-4 to extract patient concept features from the Oxford Textbook of Medicine. After manually annotating the concept features for quality, we obtain a set of 254 concept features. Next, we convert the patient clinical notes into a concept vector representation using an agreement model. The agreement model is an LLM that takes an input pair consisting of a patient note and a concept feature and outputs either a “Yes,” “Uncertain,” or “No” depending on whether the patient exhibits the corresponding feature in their note. Finally, we train a linear classification model on the concept vector representation of the clinical notes along with the gold labels (representing the dementia type) and evaluate the model on a held-out test-set. A more detailed description of each step is discussed in the following sections.

### 2.1 Dementia classification dataset

We use MIMIC-III (Johnson *et al.* 2016) to create a dataset of clinical notes from patient admissions with three different dementia types using a set of ICD-9 (International Classification of Diseases, Ninth Revision) codes (listed in Supplementary Table S1), namely *Alzheimer’s Dementia*, *Vascular Dementia*, and *Other Dementias* along with a *No Dementia* control group. The distribution of the dataset is shown in Table 1. For each patient admission, we select only the discharge summary note. Additionally, we remove the phrases *“dementia*,*” “alzheimer’s*,*” “vascular dementia*,*”* and other variants from the patient notes to prevent information leakage.

**Table 1.** Label distribution of dementia dataset created using MIMIC-III.

Label	Number of patient admissions
Vascular dementia	213
Alzheimer’s dementia	1087
Other dementia	2650
No dementia (control group)	3500

### 2.2 Creating concept features for dementia classification

We extract features related to Dementia from the Oxford Textbook of Medicine (Warrell 2003) using GPT-4 (Achiam *et al.* 2023). We first identify the chapter *Alzheimer’s Disease and Other Dementias* and extract paragraphs under the *Clinical Features* subheading. Next, we use GPT-4 to extract clinical features from the textbook paragraphs using the **Concept Extraction Prompt**. The **condition** in the prompt refers to the type of dementia described in the textbook (Frontotemporal Dementia, Vascular Dementia, etc.).

An example of an extracted sentence using the ***Concept Extraction Prompt*** is *“The patient exhibits agitation, restlessness, wandering, and dis-inhibition.”* This example contains multiple clinical features, but we ideally want each concept feature to be an atomic clinical feature. We use GPT-4 to achieve this by using the ***Concept Simplification Prompt*** which contains two input–output examples for the task (2-shot learning). Brown *et al.* (2020) showed that LLMs are proficient at in-context learning and that their few-shot performance is often much higher compared to the zero-shot setting. The example containing multiple clinical features mentioned above is broken into the following concept features containing atomic clinical features:

1. The patient exhibits agitation.
2. The patient exhibits restlessness.
3. The patient exhibits wandering.
4. The patient exhibits dis-inhibition.

*Concept Extraction Prompt*

Rewrite the following description about {**condition**} as multiple bullet points in the format “The patient experiences …”. Be as exhaustive as you can.

{**textbook description**}

The patient experiences:

*Concept Simplification Prompt*

Rewrite this sentence as multiple simple sentences beginning with “The patient exhibits” one per line.

Sentence: The patient exhibits cognitive slowing plus impairment of executive (planning and organizational abilities) and visuoperceptual abilities.

Output:

The patient exhibits cognitive slowing.

The patient exhibits impairment of executive (planning and organizational) abilities.

The patient exhibits impairment of visuoperceptual abilities.

Sentence: The patient exhibits ventricular enlargement disproportionate to the degree of cortical atrophy.

Output: The patient exhibits ventricular enlargement.

The patient exhibits cortical atrophy.

The patient exhibits ventricular enlargement disproportionate to the degree of cortical atrophy.

Sentence:{**sentence**}

Output:

We obtained 294 unique concept features which were then manually annotated for quality. Some of the extracted concept features don’t make sense due to the way we force the output to be formatted (*“The patient exhibits thalamus”* and *“The patient exhibits perfusion MRI, MRS, and PET scans may enhance diagnostic accuracy.”*). These were excluded from the final concept feature list. We also rephrased certain concept features to exhibit proper grammar (*“The patient exhibits complaints of ‘loss of memory for words’.”* was changed to *“The patient complains of a loss of memory for words.”*) and added additional information to reduce ambiguity (*“The patient exhibits inconsistency in test performance.”* was changed to *“The patient exhibits inconsistency in MMSE test performance.”*). We finally end up with 254 concept features (shown in Supplementary Table S2) that we use for the following experiments where we predict patient dementia type using clinical notes from their EHRs.

### 2.3 Converting clinical notes to concept feature vectors using agreement models

The goal of this task is to convert a patient clinical note into a vector representation where the index corresponds to one of the 254 concept features extracted and the value at that index corresponds to either a “Yes,” “No,” or “Uncertain” depending on whether that feature was present or absent (Fig. 1). To evaluate this task, we pick 10 concept features and randomly sample 20 clinical notes for each feature. We manually annotate this set containing *(patient note, concept feature)* pairs to evaluate the subsequent models used (refer to Table 6 for the selected concept features).

**Table 6.** Performance metrics across different concept features while using only GPT-4 on the manually annotated test-set.^a^

Concept feature	Concept accuracy	Concept F1
Fluctuating cognitive performance	0.85	0.83
Difficulty answering questions	0.79	0.72
Reduced speech output	0.94	0.92
Forgetfulness	0.5	0.47
Bladder dysfunction	0.85	0.80
White-matter changes	0.90	0.88
Delirium	0.89	0.87
A loss of libido	1.0	1.0
Substance dependence	1.0	1.0
Low energy	0.89	0.88

a Each concept feature is of the form *“The patient exhibits ….”*.

#### 2.3.1 Using GPT-4 to extract concept features from patient notes

We provide a prompt containing *(patient note, concept feature)* pairs to GPT-4. An example of the prompt for a specific concept feature (Optic Atrophy) is shown in the **Patient Note Concept Activation Prompt**. The definitions for the concept features were generated by GPT-4 on a separate run. Example outputs for this prompt are shown in Table 2. For every patient note, we ran this for each of the 254 concept features, obtaining a “yes,” “no,” or “uncertain.” In this manner, we built a concept vector representation of each note which we then used to train a linear classification model on to predict dementia type for a particular patient.

**Table 2.** GPT-4 outputs over different responses for the concept feature “*Optic Atrophy*” using the Patient Note Concept Activation Prompt.

Feature name	Response	Supporting information
Optic atrophy	No	None
Optic atrophy	Uncertain	Ophthamology was consulted and they recommended following her decreased visual acuity in her R. eye. Bone fragment seen in R optic strut concerning for injury to the adjacent optic nerve.
Optic atrophy	Yes	28yo F w/diabetes insipidus, diabetes mellitus, optic atrophy and deafness, and a history of multiple pneumonias…

#### 2.3.2 Reducing the number of calls to LLMs using text embeddings

A major technical challenge that we encounter is the number of calls we have to make to GPT-4 to annotate all combinations of (patient note, concept feature) pairs. Our dataset includes 7450 patient notes with 254 concept features, having a total of 1.9 million (7450×254) note-concept pairs and 7.3 billion tokens. To annotate the dataset using our access to GPT-4, it took over 50 days. In order to reduce the processing time, we investigate the use of text embeddings to minimize the number of note-concept pairs annotated by GPT-4. Rather than annotate all the pairs, we want GPT-4 to only annotate notes that are likely to have the corresponding concept activated. This is because some concepts are less common than others, occurring in only a fraction of the patient notes.

**Figure 2.**
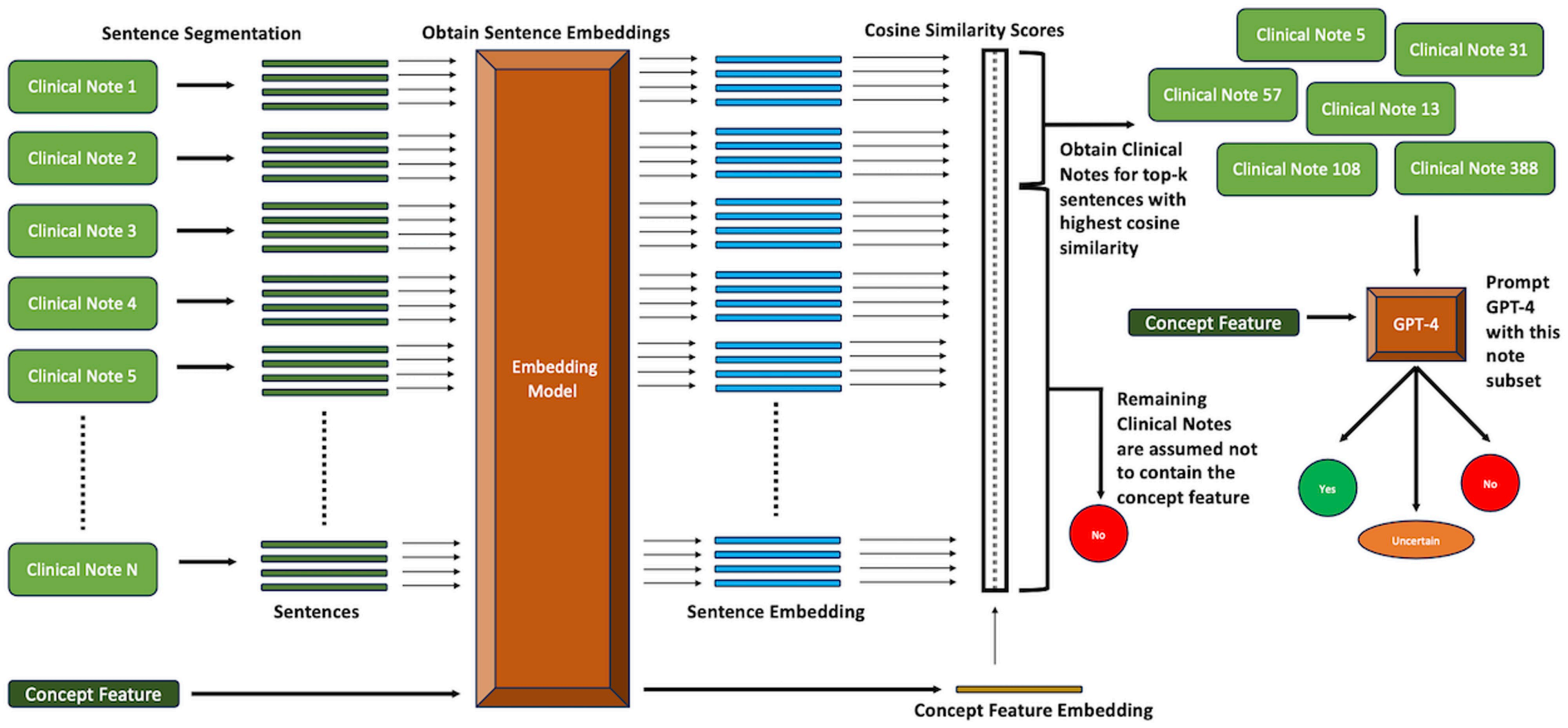
Pipeline illustrating the Text Embedding approach with GPT-4 for identifying the presence of concepts in patient notes.

An overview of our text embedding approach is shown in Fig. 2. We first tokenize the clinical notes into sentences using Stanza (Qi *et al.* 2020). Next, we embed each note sentence and each concept feature using an embedding model. We run six different sets of experiments, each with a particular embedding model (one of OpenAI’s embedding models or INSTRUCTOR models (Su *et al.* 2022) shown in Table 4). For the concept features, we append the corresponding definition generated by GPT-4 before embedding it. The INSTRUCTOR model generates contextual embeddings for text by incorporating task-specific instructions. Appending the task type before the text helps the model understand the context and nuances of the specific task, thereby improving the relevance and accuracy of the embeddings produced. As a result, for the INSTRUCTOR models, we embed each patient note sentence after prepending the phrase “*Represent the clinical note sentence for patient feature identification:*” and every concept feature after prepending the phrase “*Represent the patient feature for clinical note feature identification:*”.

**Table 4.** Performance of the Text Embeddings approach with GPT-4 using different embedding models. The bolded text represents the best performing Text Embedding approach.

Approach	Embedding model	Concept accuracy	Concept F1
Majority baseline	–	0.65	0.51
GPT-4	–	0.86	0.84
Text embeddings with GPT-4	instructor-base	0.71	0.63
Text embeddings with GPT-4	instructor-large	0.72	0.65
Text embeddings with GPT-4	instructor-xl	0.71	0.63
Text embeddings with GPT-4	text-embedding-ada-002	0.72	0.65
Text embeddings with GPT-4	text-embedding-3-small	0.72	0.64
**Text embeddings with GPT-4**	**text-embedding-3-large**	**0.73**	**0.66**

***Patient Note Concept Activation Prompt***

SYSTEM PROMPT:

You are a clinical research assistant. For the given clinical note, answer the question (“Concept Question”) provided in the dictionary. Use “Definition,” “Possible Responses,” “Response Guide,” and “Additional Response” to help better answer the question. Your response should be of the following format: “Response”: Either “Yes,” “No,” or “Uncertain,” “Supporting Information”: If “Response” is either “Yes” or “Uncertain,” provide supporting sentences from the clinical note. If “Response” is “No,” this should be “None”

CHAT HISTORY: {**clinical_note**}

USER MESSAGE:

Feature Name: Optic atrophy

Concept Question: Can the following sentence be inferred from the clinical note?: “The patient exhibits optic atrophy.”

Definition: Optic atrophy in a clinical setting refers to the damage or degeneration of the optic nerve, which transmits visual information from the eye to the brain. This often results in a variety of visual symptoms, including blurred vision, difficulties with color vision, and reduced visual field. The causes of optic atrophy can include glaucoma, optic neuritis, stroke, or trauma to the optic nerve. Optic atrophy is typically diagnosed through comprehensive eye exams and imaging studies. If left untreated, it can lead to permanent loss of vision, hence prompt diagnosis and management is essential.

Possible Responses: Yes, No, Uncertain

Response Guide: Yes: The clinical note explicitly mentions that the patient has the clinical feature. No: The clinical note does not explicitly mention that the patient has the clinical feature and there is also no information that might suggest the patient has the clinical feature. Uncertain: The clinical note contains some information that might suggest the patient has the clinical feature (through correlations), but it is not explicitly stated:

Next, we perform pairwise cosine-similarity and rank notes containing sentences with the highest similarity values for each concept feature. Finally, we select the top-k clinical notes for each concept feature and prompt GPT-4 as mentioned in Section 2.3.1. We assume that for a particular concept feature, the notes not in the top-k do not contain it.

Since smaller note chunk sizes lose information about surrounding context, we experiment with varying note chunk sizes (chunk sizes containing 1, 2, 3, 4, and 5 sentences). We also try two different sampling approaches for selecting the top-k notes. In the first sampling approach, we consider a constant top 10% of clinical notes with sentences having the highest cosine similarity values for every concept feature. However, since some concept features occur at a higher frequency than others, selecting a constant top-k (10%) for every concept feature might not be ideal. As a result, in the second sampling approach, we first select a random subset containing 300 patient notes and use GPT-4 to predict the presence of each concept feature. Based on the frequency of these concept features in the 300 note subset, we extrapolate to determine the approximate number of patient notes in the entire dataset that will contain a concept feature. For example, if the concept feature “*The patient exhibits multiple lesions*” occurs 60 times in the 300 note subset, then roughly 1500 patient notes will contain it in the entire 7450 dementia dataset.

#### 2.3.3 Using Llama2 to extract concept features from patient notes

Despite GPT-4’s high performance across various tasks, their limited accessibility and high costs make them less attainable for individuals and smaller organizations. The cost of using GPT-4 as of June 2024 for our dataset containing 7.9 billion input tokens and 59 million output tokens is $240 540 ($30/1M input tokens and $60/1M output tokens). OpenAI also provides the option of using their batch processing API at 50% of the cost (which is still significant), with a response time of under 24 h. Although the cost of GPT models is trending downward, open-source models are free to use. Therefore, we explore finetuning Llama2 for this task through knowledge distillation using a subset of GPT-4’s outputs. Knowledge Distillation is a method for transferring advanced capabilities from leading proprietary LLMs such as GPT-4 to their open-source counterparts like Llama (Xu *et al.* 2024). Researchers have used different distillation techniques, closing the performance gap between proprietary and open-source models (Chiang *et al.* 2023, Xu *et al.* 2023a).

We first use GPT-4 to create a training set of 125 000 (patient note, clinical feature) pairs with the label (Yes, No, Uncertain). Next, we utilize these examples to train a 7B and 13B Llama2 model (Touvron *et al.* 2023) using LoRA (Hu *et al.* 2021), 4-bit precision and gradient accumulation, and evaluate their performances on the manually annotated test-set. As the Llama2 models used have fewer parameters (7 Billion, 13 Billion) compared to GPT-4 (speculated to be over 1 Trillion), we expect the performance of these open-source models to be lower (Open-source models are also making advances. For example, Llama3 was released in April 2024: https://ai.meta.com/blog/meta-llama-3/).

### 2.4 Dementia classification

We train models on different representations of the patient notes to predict the dementia type (Table 1). We divide our dataset containing 7450 patient admissions into a train/val/test (0.9/0.05/0.05) split using which we train and evaluate these models.

#### 2.4.1 Logistic regression baseline using ngrams

As a baseline, we train a Logistic Regression Model using ngram representations of the clinical notes which contain unigram, bigram, and trigram features and predict the patient dementia type (Table 1). Each element in the ngram vector representation is binary, representing the presence or absence of a corresponding ngram feature.

#### 2.4.2 GPT-4 baseline

We provide the patient clinical notes to GPT-4 with the **GPT-4 Baseline Prompt** and expect one of the four label outputs: *no dementia*, *dementia*, *alzheimer’s dementia*, or *vascular dementia*.

***GPT-4 Baseline Prompt***

For the given patient clinical note, chose one of the following diagnosis labels:

no dementia

dementia

alzheimer’s dementia

vascular dementia

You should only reply with the diagnosis label and nothing else.

{**clinical_note**}

#### 2.4.3 Our LLM-aided feature engineering approach

Using the predictions from the agreement model across the entire Dementia dataset, we convert patient clinical notes into concept vectors where each vector element represents a unique concept feature. Next, we train a Logistic Regression model on these feature vectors and predict the dementia label type (Table 1).

## 3 Results and discussion

### 3.1 Creating concept feature vectors using agreement models

The agreement model is an LLM that takes an input pair consisting of a patient note and a concept feature, and outputs either a “Yes,” “Uncertain,” or “No” depending on whether the patient exhibits the corresponding feature in their note. We evaluate the agreement model using the manually annotated test-set mentioned in Section 2.3.

We first evaluate the performance of using only GPT-4 on the manually annotated test-set of (patient note, concept feature) pairs. We run two different sets of experiments, one where we prompt GPT-4 (**Patient Note Concept Activation Prompt**) without the *Definition* and the other where the *Definition* is included. The results of the manually annotated test-set are shown in Table 3 where we also report a random baseline and a majority baseline for comparison. The fine-tuned Llama2 models (Table 3) perform significantly worse compared to the baselines and are therefore less efficient even with lower costs compared to GPT-4.

**Table 3.** Performance of the agreement models compared to a random and majority baseline on the manually annotated test-set for the task of predicting concept presence in a patient note. Bolded text represents the best performing approach.

Approach	Concept accuracy	Concept precision	Concept recall	Concept F1
Random baseline	0.31	0.45	0.31	0.35
Majority baseline	0.65	0.42	0.65	0.51
Llama2-7B	0.43	0.62	0.43	0.38
Llama2-13B	0.47	0.68	0.48	0.5
GPT-4 (prompt does not contain concept definition)	0.85	0.86	0.82	0.83
**GPT-4 (prompt contains concept definition)**	**0.86**	**0.87**	**0.86**	**0.84**

**Figure 3.**
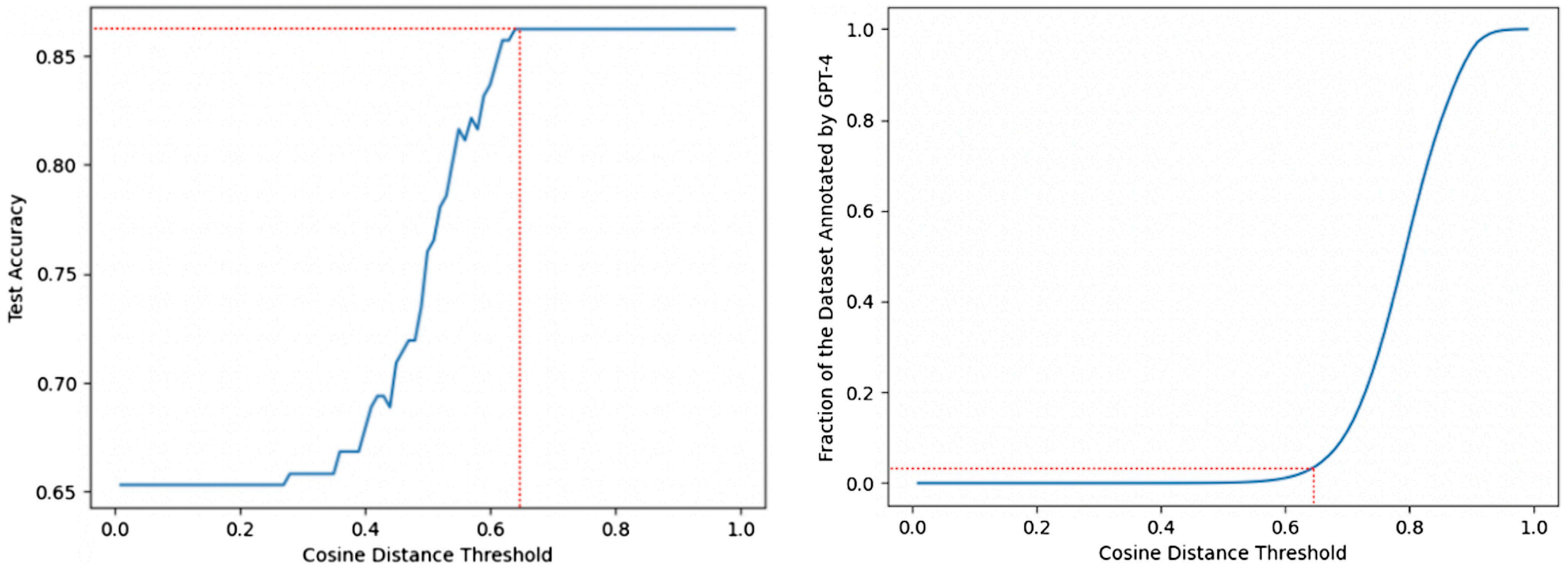
Cosine distance threshold versus the held-out test-set accuracy and the fraction of the dataset annotated by GPT-4 for the Text Embedding approach. The dotted red line indicates the cosine distance threshold at which maximum test-set accuracy is first achieved and the corresponding Dataset Fraction needed to be annotated by GPT-4.

For the Text Embedding approach, we first evaluate the different embedding models using a note chunk size of 1-sentence and a constant top-k sampling of 10% (we select the top 10% of clinical notes containing sentences with the highest cosine similarity for every concept feature). The results of these experiments on the manually annotated test-set are shown in Table 4. From the results, the OpenAI *text-embedding-3-large* model outperforms the other models by a significant margin. Next, we evaluate the two different top-k sampling approaches mentioned in Section 2.3.2 along with different note chunk sizes, shown in Table 5. The top-k sampling approach using extrapolation improves the overall performance on the test-set suggesting that in a significant portion of the (note, concept) pairs, the absence of a concept in a note can be approximated by the cosine similarity of their embeddings. Additionally, we plot the cosine distance threshold against both the test-set accuracy and the dataset fraction annotated by GPT-4, which is shown in Fig. 3. Setting a distance threshold of 0.64 achieves an accuracy of 0.86 on the development-set while requiring only 3% of the entire dataset annotated by GPT-4. With the Text Embedding approach, we can obtain a comparable performance to using GPT-4 on the entire dataset, while only requiring a fraction of the GPT-4 calls and reducing the cost from $240 540 (Section 2.3.3) to $7216.

**Table 5.** Evaluating different top-k sampling methods and different note chunk sizes for the Text Embeddings with GPT-4 approach on the manually annotated test-set. The bolded text represents the best peforming approach.

Top-k sampling approach	Number of sentences in note chunk	Concept accuracy	Concept F1
Constant (10%)	1	0.73	0.66
Varying (using extrapolation)	1	0.81	0.76
**Varying (using extrapolation)**	**2**	**0.82**	**0.78**
Varying (using extrapolation)	3	0.78	0.73
Varying (using extrapolation)	4	0.81	0.78
Varying (using extrapolation)	5	0.78	0.74

#### 3.1.1 Error analysis for concept identification in clinical notes

**Figure 4.**
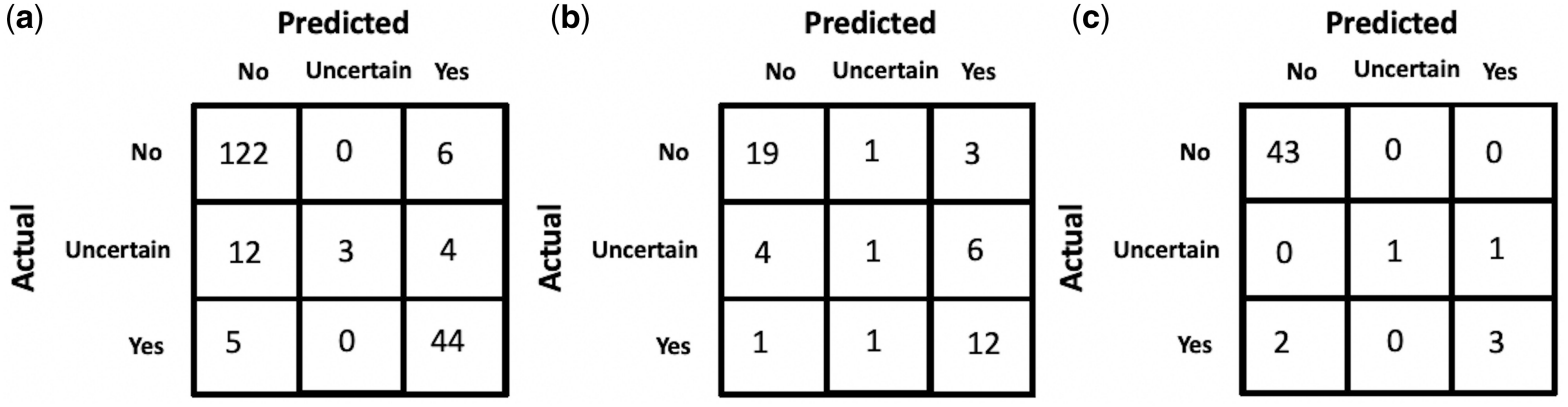
GPT-4 confusion matrices for the task of predicting if a concept is present in a clinical note (a) across all concepts in the manually annotated test-set, (b) for the concept *“The patient exhibits forgetfulness.”*, (c) for the concept *“The patient exhibits substance dependence.”*.

Among all the approaches, using GPT-4 to annotate the entire dataset performs best on the manually annotated test-set with an accuracy of 0.86. A more detailed analysis of GPT-4’s performance across different concepts in the test-set is shown in Table 6. The confusion matrices of GPT-4 for the task of predicting the presence of a concept in a clinical note are shown in Fig. 4, including results for (a) the entire test dataset, (b) across the single concept *“The patient exhibits forgetfulness.”*, and (c) across the single concept *“The patient exhibits substance dependence”*. For the individual concepts (b) and (c), we manually annotate an additional set of notes to ensure statistical significance in the confusion matrix.

For certain concepts like “*The patient exhibits substance dependence*,” physicians often explicitly state the presence/absence of the corresponding concept in the note (e.g. “*patient does not smoke and no EtOH use*” and “*patient drinks 2 beers a day….smokes marijuana daily.*”), making it easier for GPT-4 to arrive at the correct answer with little to no room for uncertainty. While GPT-4 performs well on most concept features in the test-set, some features like “*The patient exhibits forgetfulness*” and “*The patient exhibits bladder dysfunction*” perform relatively poorly. Manually reviewing some of the misclassified examples (shown in Table 7), we find that GPT-4 fails to draw logical references and has a tendency to overestimate or underestimate the correlation between a concept feature and information provided in the clinical notes (also depicted by the confusion matrices in Fig. 4 where an *“Uncertain”* gold label is often predicted as *“No”* or *“Yes”*), while in some cases completely missing vital information. For example, if a patient was stated to be disoriented, GPT-4 predicted that the patient exhibited forgetfulness which is not always an outcome that co-occurs with disorientation. Similarly, a patient who does not know her home medications is not necessarily forgetful but rather, could be ignorant of the fact. There were some examples where GPT-4 missed important information in the patient notes. For instance, a 74-year-old with a fever of 104, malaise, nausea, and vomiting is very likely to exhibit low energy while another patient with mild cognitive impairment (MCI) who is oriented to place but not year is very likely to exhibit delirium (Table 7). While the overall performance on the test-set was high, the examples in Table 7 highlight some weaknesses in GPT-4’s comprehension of clinical text.

**Table 7.** Misclassified GPT-4 examples for Concept prediction from patient clinical notes.^a^

Concept feature	Gold label	Predicted label	Explanation
Forgetfulness	Uncertain	Yes	GPT-4s explanation: “The clinical note mentions: ‘You were disoriented during your stay and we had to utilize soft restraints …… Mental status is partially oriented…’ These instances suggest disorientation and potential issues with memory, which could be indicative of forgetfulness.”
Forgetfulness	Uncertain	Yes	GPT-4s explanation: *“Pt did not know her home medications”*.
Low energy	Uncertain	No	The clinical note includes the following information that might correlate with low energy: *“The patient is a 74-year-old … who presented to the Emergency Department with a fever of 104, malaise, nausea, and vomiting…”*. This was missed by GPT-4
Difficulty answering questions	Yes	No	The clinical note includes the following information that might correlate with difficulty answering questions: *…Upon awakening and extubation, patient was slow to answer questions and mildly inattentive (but this improved next day). Mental status was otherwise normal. …*. This was missed by GPT-4.
Delirium	Yes	No	The clinical note contains the following information that might suggest delirium: *“Pt is oriented to place ‘hospital’, but not year. She was not sure why she is here. Per family, pt does have some mci and this is baseline for pt.”*. This was missed by GPT-4.

a Each concept feature is of the form *“The patient exhibits ….”*.

### 3.2 Dementia classification

**Figure 5.**
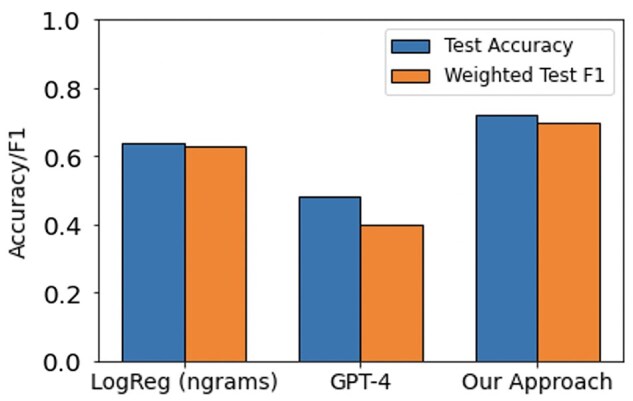
Accuracy and F1 scores on a held-out test-set for two baselines (LogReg and GPT-4) and our approach for the task of predicting dementia type for a patient.

**Figure 6.**
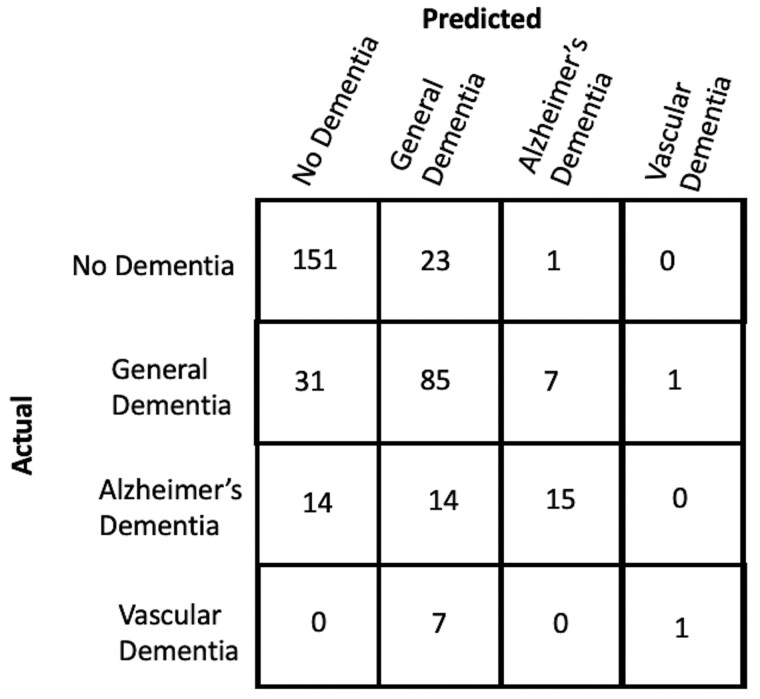
Confusion matrix across different labels in the held-out test-set for our approach.

Results of the various agreement model approaches are provided in Tables 3–5. While Llama 13B outperformed Llama 7B in our prediction example, it still did not surpass the majority baseline in terms of F1-score. Therefore, we did not pursue using this method further despite its inexpensive cost. As GPT-4 achieves the highest accuracy and F1 scores at identifying concept features in patient notes, we use it to construct concept vector representations for every note in the dementia dataset. We train and evaluate a Logistic Regression model on this representation to predict what type of dementia (if any) a patient has. This is compared to the performance of two different baselines: (i) an ngram-Logistic Regression model trained on unigram, bigram, and trigram representations of the patient notes, and (ii) a GPT-4 baseline (described in Section 2.4.2). The top features of the ngram-Logistic Regression model for each label are shown in Table 8. The performance of both these baselines and our approach on a held out test-set is shown in Fig. 5. A more detailed set of results are provided in Supplementary Table S3. Our approach significantly outperforms the other baselines across both accuracy and F1 scores. The confusion matrix across the different classes for our approach is shown in Fig. 6.

**Table 8.** The top ngram features for the baseline Logistic Regression model for different class labels.

No dementia	Other dementia	Alzheimer’s dementia	Vascular dementia
weeks	care	cxr	Per
date	facility	died	Were
pain	heparin	the patient	But
discharge	mild	he	aspiration
infant	htn	aricept	hospital
birth	likely	hemorrhage	Ed
good	started	but	vancomycin
lung	baseline	iv	the hospital
tube	there is	baseline	per day
remained	expired	risk	neurology

#### 3.2.1 Analyzing most important features for dementia prediction

We rank the most important features for each dementia-type label according to the weights of the trained Logistic Regression models. While some of the top ngram features such as *heparin, htn (hypertension)*, and *aricept* can be indicative of a patient having dementia, several others are not clinically meaningful features to predict dementia type (Table 8). For instance, the presence of ngrams such as *cxr, died, the patient, he, iv, baseline* in the clinical notes should not be significant contributors in diagnosing Alzheimer’s Dementia for a patient. Such models should therefore be used with caution in the clinical setting as the underlying decision-making process does not align with that of a healthcare professional.

The top predictive features for our approach are shown in Table 9. For vascular dementia, the most predictive feature was *“memory loss that may be less prominent than in Alzheimer’s Disease*.*”* This is supported by Mayo Clinic (https://www.mayoclinic.org/diseases-conditions/vascular-dementia/symptoms-causes/syc-20378793) which states that memory loss is not a significant symptom of Vascular Dementia compared to Alzheimer’s Dementia. Additionally, *infarcts involving multiple main arterial territories* (McKay and Counts 2017) and *history of atherosclerotic risk factors* (Nordestgaard *et al.* 2022) have been shown to be clinical indicators of vascular dementia. Karantzoulis and Galvin (2011) mention that semantic fluency is comparatively better preserved in Vascular Dementia compared to Alzheimer’s Dementia, supporting our results for *poor performance on semantically based tasks* being a negative predictive feature for Vascular Dementia and *profound loss in conceptual knowledge (or semantic memory)* being a top positive predictor for Alzheimer’s disease. Looking at the top features for the control group (No Dementia), “*progressive cognitive decline*,” “*cognitive impairment*,” and “*poor memory*” are negatively correlated as expected.

**Table 9.** Top features of the Logistic Regression model trained on concept vector representations of the patient notes for different class labels.

No dementia	Other dementia	Alzheimer’s dementia	Vascular dementia
Progressive cognitive decline **(No)**	Poor performance on semantically based tasks **(Yes)**	Memory loss that may be less prominent than in Alzheimer’s disease **(No)**	Memory loss that may be less prominent than in Alzheimer’s disease **(Yes)**
Cognitive impairment **(No)**	Impaired comprehension of words **(Yes)**	Decreased reflexes **(Yes)**	Narrowing of the vessel **(No)**
Poor memory **(No)**	Amnesia **(No)**	Profound loss in conceptual knowledge or semantic memory **(Yes)**	Infarcts involving multiple main arterial territories **(Yes)**
Poor concentration **(Yes)**	Myoclonus **(No)**	Rapid onset of memory impairment **(No)**	Poor performance on semantically based tasks **(No)**
History of atherosclerotic risk factors **(No)**	Bladder dysfunction **(Uncertain)**	Mental inflexibility **(Yes)**	History of atherosclerotic risk factors **(Yes)**
Rigidity **(No)**	Poor concentration **(No)**	Progressive cognitive decline **(Yes)**	Forgetfulness **(No)**
Profound loss in conceptual knowledge or semantic memory **(No)**	Profound loss in conceptual knowledge or semantic memory **(Uncertain)**	Fluent speech **(Uncertain)**	Apathy **(No)**
Amnesia **(Uncertain)**	Large infarcts **(Yes)**	Visuospatial difficulties **(Yes)**	Poor performance on neuropsychological tests **(No)**
Substance dependence **(Uncertain)**	Reduced speech output **(Uncertain)**	Lack of initiation **(Uncertain)**	Large infarcts **(Uncertain)**
Subcortical dementia syndrome **(No)**	Fluent speech **(Yes)**	Severe forgetfulness **(Yes)**	Restlessness **(No)**

Each cell contains a concept of the form *“The patient exhibits—”*. **(Yes)** indicates the patient exhibits the corresponding clinical feature, **(No)** indicates the patient does not exhibit the corresponding clinical feature and **(Uncertain)** indicates the patient may or may not exhibit the corresponding clinical feature.

The top features of our LLM-aided feature engineering approach involve high-level clinical indicators that were extracted from the Oxford Textbook of Medicine. These might overlap with some of the more important clinical features that trained healthcare professionals focus on during the diagnosis of dementia type for a patient, thereby reducing the likelihood of spurious correlations.

### 3.3 Limitations and future work

An advantage of our LLM-aided feature engineering approach to predict patient dementia type is its interpretability while also being accurate. The results shown in Fig. 6 suggest that there is still potential for improvement in classifying Alzheimer’s and Vascular Dementia, which could be achieved by addressing several limitations. A limitation of this study lies in the challenges of accurately identifying clinical features within patient notes, as GPT-4 sometimes fails to interpret certain clinical details. This issue might result from the forced phrasing of clinical features and the format of the prompt used. Additionally, this study only considers clinical features relevant to predicting dementia, omitting nonclinical factors such as demographics and lifestyle which are crucial for comprehensive patient assessments. The absence of temporal elements on the onset and progression of clinical features also limits the model’s ability to fully capture disease trajectories. Moreover, due to computational constraints, the study focuses on a limited set of clinical features which may hinder the generalization of the approach. The model was also evaluated solely on data from a single hospital, which may not reflect variations across different institutions. Keeping these limitations in mind, future work should focus on integrating nonclinical features, incorporating temporal information, expanding to a broader set of clinical features, and an evaluation across multiple hospitals.

## 4 Conclusion

In this article, we presented a novel approach for predicting dementia type from patient clinical notes using an LLM-aided feature engineering approach. Our key contributions were:

1. Developing a method to extract clinically relevant concept features related to dementia from the Oxford Textbook of Medicine using GPT-4. This resulted in 254 interpretable concept features like “*The patient exhibits memory loss*” and “*The patient complains of forgetfulness.*”
2. Converting patient clinical notes into concept vector representations using Agreement Models, which predict whether each concept feature is present, absent, or uncertain based on the note text.
3. Training an interpretable linear classification model on the concept vectors to predict dementia type, achieving 0.72 accuracy that outperformed an ngram baseline (0.64) and GPT-4 (0.48).
4. Analyzing the important concept features and finding that top predictors aligned with clinical understanding, like memory loss being less prominent for vascular dementia compared to Alzheimer’s.

Our approach forced the model to focus on high-level clinically relevant concepts during prediction, potentially reducing hallucinations and spurious correlations compared to opaque black-box models. Interpreting the important concept features provided insights into the model’s reasoning process. However, there were some limitations. Agreement models like GPT-4 still sometimes failed to comprehend clinical details, highlighting the need for further improvements.

Overall, the framework in this paper offers an interpretable and accurate paradigm for clinical prediction tasks like dementia classification. Future work could explore extending this to other disease areas and using temporal information for clinical features. Ensuring reliable and trustworthy AI systems will be crucial for robust clinical deployment.

## Supplementary Material

btaf156_Supplementary_Data
