# Supplementary material for: Predicting explainable dementia types with LLM-aided feature engineering
Source: Bioinformatics. 2025 Apr 8;41(4):btaf156. doi: 10.1093/bioinformatics/btaf156 (PMC12021793; doi:10.1093/bioinformatics/btaf156)
Supplement: btaf156_Supplementary_Data [file btaf156_supplementary_data.pdf]

Predicting Explainable Dementia Types with LLM-aided Feature Engineering: **Supplementary Material**

Aditya Kashyap Delip Rao Mary Regina Boland Li Shen

Chris Callison-Burch

1 Dementia Classification Dataset

We use the ICD-9 codes listed in Table 1 to automatically obtain patient admissions for different dementia types in the MIMIC-III dataset.

Table 1: ICD-9 Codes Used to identify patient admission dementia types

**Classification Label**	**ICD-9 Codes**
No Dementia	-
Vascular Dementia	290.40, 290.41, 290.42, 290.43
Alzheimer's Dementia	331.0
Other Dementia	290.0, 290.10, 290.11, 290.12, 290.13, 290.20, 290.21, 290.3, 291.2, 292.82, 294.10, 294.11, 294.2, 294.21, 331.19, 331.82

2 Concept Features Extracted from GPT-4

The entire set of extracted clinical concepts from the Oxford Textbook of Medicine using GPT-4 is shown in Table 2.

peripheral mass lesions	relatively unaffected visuospatial function	delayed verbal recall
reduced speech output	non-fluent speech	biological features of depression
pleocytosis	diminished vibration sense	recurrent cerebral infarcts
dyspraxia	change in food preference	impaired naming with semantically based errors
fluctuating cognitive performance	difficulty with complex visuospatial tasks	abnormalities in the parietotemporal regions on SPECT scans
psychomotor slowing	rapid shifts between hypoactivity and hyperactivity	disturbance of the sleep–wake cycle
apathy	multiple phonological errors	speech disorder
behavioural problems	decreased reflexes	poor performance on neuropsychological tests
worsening of semantic memory	anosmia	ventricular dilatation
mood swings	apraxia	confluent subcortical and corpus callosal demyelination
poor concentration	patchy performance on memory tasks	relatively spared memory in the early stages
a loss of appetite	impaired comprehension of faces	pout reflex
delusions	reduced category fluency	stereotyped food choices
amnesia	gross impairments in the grammatical aspects of language production	visuospatial function
mental slowing	lack of subjective feelings	perceptual distortions
impaired judgement	optic atrophy	visual hallucinations
poor understanding of complex words	sensitivity to neuroleptic medication	relatively preserved day-to-day memory (episodic memory)
indifference to professional responsibilities	lobar haemorrhages	difficulty answering questions
of a loss of memory for words	illusions	narrowing of the vessel
ventricular enlargement disproportionate to the degree of cortical atrophy	mild extrapyramidal features	marital problems
mutations on chromosome 19	Babinski reflex	small-vessel disease
disinhibition	restlessness	impairment of executive abilities
financial problems	agitation	ambulatory difficulties
involuntary movements	hallucinations	perseverations
a tendency to echolalia	snout reflex	sleep disturbance
socially inappropriate behaviour	pathological changes in the cutaneous blood vessels	loss of the cerebral white matter
periventricular high-signal changes	impaired repetition	changes in personality
sensory ataxia	white-matter changes	acute onset of attentional abnormalities
poor memory	giving bizarrely wrong answers to questions	difficulty with visuospatial tasks
a lack of interest in hobbies and activities	past psychiatric history	inconsistency in mmse test performance
memory loss that may be less prominent than in Alzheimer's disease	stroke-like episodes	cognitive slowing
mild tremor	severe forgetfulness	difficulty in managing complex day-to-day activities
pout reflexes	extensor plantar responses	abnormalities in the parietotemporal regions
ganser syndrome	history of atherosclerotic risk factors	forgetfulness
increased tone in their voice	preference towards sweet foods	oligoclonal bands
insidiously progressive changes in personality and behaviour	repetitive questioning	mild tremor with postural and action components
poorly articulated speech	gait disturbance	migraine-like headaches
cognitive impairment	gross impairments in the phonological (sound-based) aspects of language production	large infarcts
poor understanding of complex concepts	impaired repetition of multisyllabic phrases	Binswanger's disease
indifference to domestic responsibilities	progressive cognitive decline	mutations in the notch3 gene on chromosome 19
confluent subcortical demyelination	a history of theft	memory loss
visuospatial impairments	subcortical dementia syndrome	increased reflexes
muscle fasciculations	infarcts involving multiple main arterial territories	a loss of libido
impaired repetition of multisyllabic words	wasting	snout reflexes
gait apraxia	multiple lacunar lesions	bladder dysfunction
multiple lesions	diffuse white-matter disease	increased protein and oligoclonal bands
well-preserved word comprehension	postural components of tremors	difficulty understanding less frequent words
incontinence	visuospatial disturbance	intracerebral haemorrhages
grasp reflex	fluent speech	word-finding difficulties
impaired comprehension of words	substance dependence	trouble with the law
rigidity	agrammatical speech	symmetrical diffuse low-density periventricular hypodensity
impairments in job performance	obsessive–compulsive behaviour	good visuospatial skills
joint contractures	sparse language output	relatively spared memory
poor performance on semantically based tasks	unawareness of the meaning of words and phrases	macrocytic anaemia
deterioration of social skills	raised intracranial pressure	subcortical infarcts
spontaneous fluctuations in cognitive abilities	mutism	greatly increased rigidity
mental inflexibility	diminished vibration sense in the lower limbs	stereotyped daily routines
action components of tremors	well-preserved object recognition	pathological changes in the cutaneous blood vessels in a skin biopsy
leucoencephalopathy (CADASIL)	anomia	neuropsychiatric symptoms
potential concomitant organic disorder	word substitutions	severe language impairment
loss of personal identity	language difficulties	mixed reflexes
periventricular hypodensity	low energy	ataxia
rapid onset of memory impairment	asymmetries on the neurological examination	shrinking vocabulary
loss of salient personal events	small lesions	focal neurological signs
impairment of visuoperceptual abilities	personality changes	poor recent memory
corpus callosal demyelination	impairment of episodic memory	impaired naming of low-frequency words
tendencies to hoard food	lack of feelings of depression	primitive reflexes such as snout, pout, and grasp
narrowing of the vessel to produce ischaemia	cortical atrophy	mutations in the notch3 gene
fleeting misidentification phenomena	profound loss in conceptual knowledge (or semantic memory)	semantically based errors
delirium	patchy performance on frontal tasks	lacunar infarcts
empty speech	jocularity	changes in behaviour
peripheral mass lesions of varying signal density on CT	extrapyramidal signs	hyperactivity
abnormal sexual behaviour	impairments in social function	disturbance of consciousness
seizures	unilateral visual failure	hypoactivity
lack of initiation	non-verbal problem-solving ability	fatuousness
marked fluctuations in cognitive abilities	impaired naming	myoclonus
pupillary abnormalities	cerebral amyloid angiopathy	worsening of language function
grasp reflexes	ventricular enlargement	delayed verbal recall of new material
impaired comprehension of objects	distractibility	frontal executive malfunction
impaired attention	periventricular high-signal changes on T2-weighted MRI	gradual loss of expressive abilities
rapid onset of intellectual impairment	cerebral autosomal dominant arteriopathy	ischaemia
reduced ability to maintain attention to external stimuli	marked fluctuations in behaviour	emotional lability
medial temporal lobe atrophy	visuospatial difficulties	psychomotor disturbance
poor performance on neuropsychological tests of frontal function	ophthaloplegia	wasting particularly affecting the bulbar musculature
normal repetition of words and phrases	inability to appropriately shift attention to new stimuli	

Table 2: 254 Clinical Features of Dementia extracted by GPT-4 from the Oxford Textbook of Medicine after manually annotating for quality. Each cell represents a concept of the form *The patient exhibits —*.

Approach	Dementia Accuracy	Dementia Precision	Dementia Recall	Dementia F1
Random Baseline	0.29	0.44	0.29	0.34
Majority Baseline	0.48	0.23	0.48	0.31
Logistic Regression with ngrams	0.64	0.62	0.64	0.63
GPT-4	0.48	0.69	0.48	0.40
Our Approach	0.72	0.71	0.72	0.70

Table 3: Performance of different approaches for dementia type classification on a held out test-set using patient clinical notes

3 Dementia Classification

The random baseline, majority baseline and performance of different approaches on a held out test-set is shown in Table 3.

4 Zero-shot vs Few-shot Prompting for GPT-4

In this paper, we use four different prompts across our experiments:

1. Concept Extraction Prompt (Section 2.2)
2. Concept Simplification Prompt (Section 2.2)
3. Patient Note Concept Activation Prompt (Section 2.3.1)
4. GPT-4 baseline Prompt (Section 2.4.2)

For prompt **1.**, we manually reviewed the outputs and noticed that 0-shot prompted GPT-4 accurately extracted patient clinical features from the medical textbook in an exhaustive manner. Hence, we did not use few-shot prompting. For prompt **2.**, we noticed that GPT-4 incorrectly simplified complex sentences such as "The patient exhibits cognitive slowing plus impairment of executive (planning and organizational abilities) and visuoperceptual abilities." and "The patient exhibits ventricular enlargement disproportionate to the degree of cortical atrophy.". As a result, we selected a 2-shot prompting approach to improve on this error and through manual review of the output, we found that the output was of high quality. For prompt **3.**, we chose 0-shot prompting over few-shot prompting for the following reasons:

- Due to the length of the patient notes, including even one example for 1-shot prompting would increase the token requirements drastically for every prompt, thereby increasing costs and run time.
- Due to the varying formats of clinical notes and the number of clinical features (254), finding a subset of (note-feature pairs) to annotate to ensure average performance gain across the dataset would be a research problem of its own, and is not trivial. This would also require a lot more manual annotation.

For prompt **4.**, we wanted to evaluate how 0-shot GPT-4 with no added context would perform for our Dementia type classification task.
